# A comparison between one- and two-fluoroscopic techniques in percutaneous vertebroplasty

**DOI:** 10.1186/1471-2474-9-67

**Published:** 2008-05-13

**Authors:** Yen-Yao Li, Tsung-Jen Huang, Chin-Chang Cheng, Robert Wen-Wei Hsu

**Affiliations:** 1 Department of Orthopaedic Surgery, Chang Gung Memorial Hospital at Chia-Yi, College of Medicine, Chang Gung University, Taoyuan, Taiwan

## Abstract

**Background:**

Percutaneous vertebroplasty (PV) is generally performed under fluoroscopic guidance. Technically, single fluoroscope is considered sufficient for effectively monitoring PV. However, single fluoroscopic technique might be time-consuming in rotating the C-arm of the fluoroscope for either antero-posterior (AP) or lateral radiographic view, and causing delay in detecting cement leakage that can occur if the correct sight is not given. The aim of the current investigation was to compare the efficacy and safety of performing PV using one or two sets of fluoroscope.

**Methods:**

This retrospective study enrolled 43 patients with painful osteoporotic vertebral fractures and they were treated with one-level PV. A single orthopaedic surgeon operated on all these patients. The patients were divided into two groups on the basis of the method of fluoroscopic control. In Group 1 (15 patients), PV was performed under the assistance of one fluoroscope. In Group 2 (28 patients), PV was performed under the control of two fluoroscopes. The mean follow-up was 19 months (range, 12 to 30).

**Results:**

Neither symptomatic cement leakage nor postoperative infection was found in both groups. The mean operation time in Group 2 was shorter, 37.8 vs. 31.0 minutes for Groups 1 and 2, P = 0.03. The incidence of cement leakage for Groups 1 and 2 was 26.7% (4/15) vs. 14.3% (4/28), respectively, P = 0.19.

**Conclusion:**

We found that the two-fluoroscopic technique can provide simultaneous, real-time AP and lateral radiographic views to monitor entry point and cement delivery for PV and therefore reduce the operation time. The two-fluoroscopic technique did not require a complex manpower organization and has been proved to be a safe and effective technique for PV.

## Background

Percutaneous vertebroplasty (PV) has gained its popularity for more than 10 years to treat painful osteoporotic vertebral fractures [1-7]. It is generally performed under fluoroscopic or combined a CT and fluoroscopic guidance [2]. However, currently in the United States fluoroscopy is noted nearly universal as a guidance method for PV [5]. The vertebral landmarks for needle insertion are well identified during the routine fluoroscopic technique. Single fluoroscope can be used for guiding needle insertion and monitoring cement injection as rotating around the C-arm for taking antero-posterior (AP) and lateral views of the spine. Since there are no AP and lateral views available simultaneously, the using of single fluoroscope lacks the real-time ability of fluoroscopy to monitor PV on both planes. Thus, it can make difficult to identify the entry point of the needle and delay to find cement leakage that may occur if the correct sight is not given.

**Figure 1:**
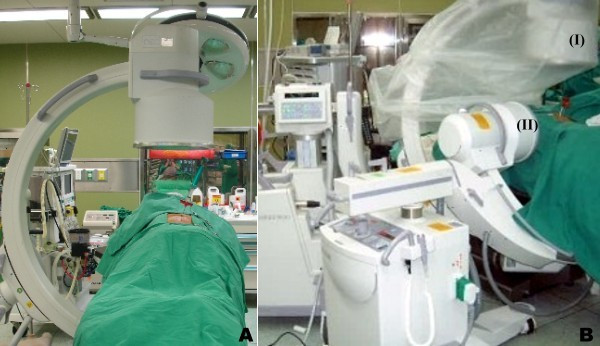
**(A) Placement of one C-arm fluoroscope, and (B) Placement of two C-arm fluoroscopes.** The AP fluoroscope (I) (GE/OEC 9800, Salt Lake City, UT, USA), was positioned vertically with the C-arm's pivot maintained at 45° or less to the long axis of the operation table. The C-arm of the lateral fluoroscope (II) (GE/OEC 7700, Salt Lake City, UT, USA) was placed under the operation table horizontally while it was tilted away from the C-arm of the AP fluoroscope to prevent collision.

The use of two sets of fluoroscope in performing PV has not been reported in the literature. The aim of the current investigation was to compare the efficacy and safety of performing PV using one or two sets of fluoroscope (Figure 1).

## Methods

Forty-three consecutive patients who underwent one-level PV by a single surgeon and followed-up for at least one-year were enrolled in the study. The indications of surgery were painful osteoporotic vertebral fractures with unsuccessful conservative treatment for at least six weeks. These patients were divided into two groups according to whether or not two fluoroscopes (GE/OEC 7700 and GE/OEC 9800, Salt Lake City, UT, USA) were available simultaneously. While one of two fluoroscopes was being used for other surgeries, such as posterior instrumentation for spinal surgeries or closed reduction for fractures etc., single fluoroscope could be only available for PV. Group 1 was composed of 15 patients who received PV under the guidance of one fluoroscope. Group 2 was composed of 28 patients who were treated under the radiographic control of two sets of fluoroscope. The mean follow-up was 19 months (range, 12 to 30). This study was approved by the Institutional Review Board of the Chang Gung Memorial Hospital (Reference number 97-0535B). The written informed consents were obtained from all the patients enrolled in the study.

Magnetic resonance imaging (MRI) with enhancement was routinely performed pre-operatively for all patients. MRI was adopted to exclude malignant vertebral fractures, and to determine the healing status of vertebral fractures, among which non-healed fractures were selected for PV.

To assess the patient satisfaction, we adopted the following questionnaire designed by Mirovsky et al. [8] to ask all patients by direct or telephone interviews at the final follow-up: "Considering the degree of pain relief following PV, would you grade your satisfaction as: 1) Very satisfied, 2) Satisfied, 3) Cannot decide, 4) Not satisfied". Independent investigators excluding the surgeon analyzed the patient satisfaction and radiographic measurements for cement leakage.

The operation time was recorded that was from the commencement of the local anesthesia to the removal of the trocar needle following the solidification of the delivered bone cement, which was mixed with Zimmer Dough type cement (40 g polymer powder and 20 ml Monomer liquid, Warsaw, IN, USA) and additional barium sulfate (5 g). Additionally, the amount of the delivered cement mixture for each vertebra was recorded.

Statistically, the differences between the two groups were examined by using the Wilcoxon rank sum test or the Fisher exact test. A P-value less than 0.05 was considered statistically significant.

### Case Illustration

**Figure 2:**
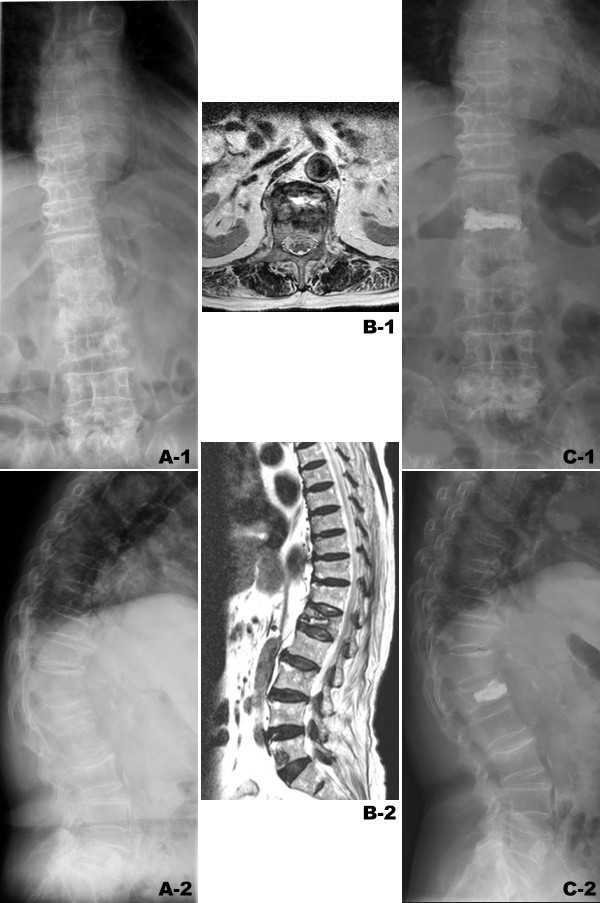
**Case no. 23 in Group 2.** A 78-year-old female patient suffered from prolonged mid-back pain secondary to a fall accident. (A-1,2) Plain radiographs showed L1 osteoporotic compression fracture. (B-1,2) the T2W MR image demonstrated non-healed L1 compression fracture as presenting high signal intensity in intra-vertebral cleft of L1 body. (C-1,2) L1 vertebroplasty was performed successfully without cement leakage. The operation time was 21 minutes. The patient was very satisfied with the procedure.

A 78-year-old female patient (Case no. 23, Group 2) had suffered from severe back pain secondary to a fall accident. The back pain was exacerbated on weight-bearing posture. Physically, there was a local tenderness over the midline of her mid-back. The imaging studies showed a L1 osteoporotic compression fracture (Figure 2). Conservative treatment including bracing was prescribed for 6 weeks. However, the symptom did not subside. Thereafter, she was advised the undergo PV with the two-fluoroscopic technique. This technique is unique in the positioning of the two fluoroscopes for the procedures. The two fluoroscopes were placed on the same side, enabling the surgeon to perform the operation comfortably on the other side (Figure 1B). The fluoroscope for taking AP radiographs (the AP fluoroscope) was placed cephalad to the fluoroscope for taking lateral radiographs (the lateral fluoroscope) according to patient's position. The AP fluoroscope was positioned vertically with the C-arm's pivot maintained at 45° or less to the long axis of the operation table. The C-arm of the lateral fluoroscope was placed under the operation table horizontally while the C-arm was tilted away from the C-arm of the AP fluoroscope to prevent collision. The entry point of the pedicle of the treated vertebra can be easily identified under the guidance of the two fluoroscopes. PV was performed successfully throughout the procedure and without a cement leakage. The total operation time was 21 minutes and the patient reported "very satisfied" with the procedure by the telephone interview 15 months after PV.

## Results

In this study, the patients' demographic and clinical data were illustrated in Tables 1 and 2. In Group 1, the treated levels were T11 (*n *= 1), T12 (*n *= 5), L1 (*n *= 5), L2 (*n *= 3) and L3 (*n *= 1). The mean amount of cement delivered was 4.3 ± 1.0 ml (range, 3 to 7). There were four patients (26.7%) with cement leakage without neurologic deficits. No superficial wound infection or infected spondylitis was noted. There was one patient with one transient subcutaneous hematoma, postoperatively. The mean operation time was 37.8 ± 6.9 minutes (range, 26 to 50). Five patients (33%) were very satisfied with the procedure, and four patients (27%) were satisfied. The other six patients (26%) reported that they were not satisfied, who claimed that there was little or no improvement in back pain. No patient complained of worse pain at the treated level, or experienced any new neurologic deficits.

**Table 1:** Patients' Characteristics

**Patient**	**Sex**	**Age (year)**	**Operation time (minute)**	**Level**	**Cement amount (mL)**	Cement leakage*	**Patient satisfaction†**
**Group 1 (one-fluoroscopic technique)**							
1	F	80	35	T12	4	Y	-
2	F	74	26	T12	3	Y	Y
3	F	68	35	T12	7	-	Y
4	F	65	35	L2	4	-	Y
5	F	65	40	T12	4	Y	Y
6	F	65	36	L2	4	-	-
7	F	79	40	L2	5	-	Y
8	F	78	40	T11	4	-	-
9	F	74	30	T12	4	-	-
10	F	62	30	L1	5	-	-
11	F	66	50	L1	5	Y	Y
12	F	71	50	L1	4	-	Y
13	F	67	35	L3	4	-	Y
14	M	78	45	L1	5	-	Y
15	F	82	40	L1	3	-	-
**Group 2 (two-fluoroscopic technique)**							
1	F	62	31	L1	4	-	Y
2	F	73	29	L1	4	-	Y
3	F	70	40	T11	5	-	Y
4	F	79	36	L1	3	-	-
5	F	74	21	T12	4	Y	Y
6	F	73	39	L1	4	-	Y
7	F	86	26	T12	3	-	-
8	F	77	36	L2	3	-	-
9	F	77	21	T12	4	-	Y
10	F	85	20	L2	4	-	Y
11	M	77	26	T11	4.5	-	Y
12	F	74	41	L1	4	-	Y
13	M	75	31	T8	2.5	Y	Y
14	F	76	32	T11	2	Y	Y
15	F	66	46	L1	4	-	Y
16	F	77	36	L3	4	Y	Y
17	M	88	22	L1	6	-	-
18	F	82	21	T12	4	-	Y
19	F	64	21	T11	4	-	Y
20	F	67	21	L4	5	-	Y
21	F	65	46	T11	4	-	Y
22	F	82	36	T12	6	-	-
23	F	78	21	L1	6	-	Y
24	F	72	36	L2	4	-	-
25	F	74	31	T9	4	-	-
26	F	67	31	L1	4	-	-
27	F	81	29	L1	4	-	Y
28	F	77	41	L4	4	-	-

Cement leakage*: (Y), cement leakage; (-), no cement leakage.

Patient satisfaction†: (Y), very satisfied or satisfied; (-), cannot decide or not satisfied.

**Table 2:** Patients' Demographic and Clinical Data

	**Group 1 (N = 15)**	**Group 2 (N = 28)**	**P*-value***
**Age (yr)**	71.6 (62~82)	74.9 (62~82)	0.22
**Sex**	1 M, 14F	3M, 25F	0.40
**Operation time (min)**	37.8 (26~50)	31.0 (20~46)	0.03
**Cement amount (mL)**	4.3 (3~7)	4.0 (2~6)	0.37
**Cement leakage (%)**	4 (26.7%)	4 (14.3%)	0.19
**Satisfaction (%)**	9 (60.0%)†	19 (67.8%)†	0.23

† The sum of patients with very satisfied and satisfied.

In Group 2, the treated levels were T8 (*n *= 1), T9 (*n *= 1), T11 (*n *= 5), T12 (*n *= 5), L1 (*n *= 10), L2 (*n *= 3), L3 (*n *= 1) and L4 (*n *= 2). The mean amount of cement delivered was 4.1 ± 0.9 ml (range, 2.5 to 6). There were four patients (14.3%) with cement leakage without neurologic deficits. No superficial wound infection or infected spondylitis was noted. The mean operation time was 31.0 ± 8.1 minutes (range, 21 to 46). Fourteen patients (50%) were very satisfied with the procedure, and five patients (18%) were satisfied. The other nine patients (32%) reported that they were not satisfied. No patient complained of worse pain at the treated level, or experienced any new neurologic deficits.

Statistically, the operation time in Group 1 with that in Group 2 (Table 2), the differences were significant (P = 0.03). There were no significant statistical differences in the incidence of cement leakage between Group 1 and Group 2 (Table 2).

## Discussion

In the current study, we found that the median operation time in Group 2 with a two-fluoroscopic assistance was shorter than that of Group 1, 31.0 vs. 37.8 minutes, P = 0.03. Such a technique provides an immediate intraoperative biplanar image acquisition and therefore proved to be an excellent surgical guidance for safe needle insertion and cement delivery. With the assistance of concurrent AP and lateral views of fluoroscopes, the entrance site is settled easily without waiting for rotating the C-arm for the other view. Apart from the shorter median operation times in Group 2 patients, the other important issue was that surgeon may feel more comfortable and bear no fear of whether the cement leakage happening at the other view or not.

The difference in the incidence of cement leakage between both groups was not significant; the incidences were 26.7% in group 1 and 14.3% in group 2. To decrease the risk of cement leakage in Group 1, one must slowly inject cement into the vertebral body and wait a while to rotate the C-arm for checking the other view after every bolus of cement delivery. It is part of reason why the operation time was longer in Group 1 than that in Group 2. We believe that the low incidence of cement leakage deserves the longer operation time. This study revealed that using two-fluoroscopic technique, the incidence of cement leakage (14.3%) was lower than other studies [8-12] (20–58%), which used various radiographic guidance methods including single fluoroscopic or combined CT and fluoroscopic guidance.

Because spinal canal compromise resulting from leaked bone cement can cause devastating complications [13-16], cement delivery was ceased as soon as the stream of cement approached the posterior vertebral wall on the fluoroscope. Cement leakage into paravertebral tissues is commonly reported in the literature [2,10-12,17], and most of them were asymptomatic. However, paravertebral cement leakage could result in lumbar radiculopathy or intercostal neuralgia if it travels by way of neural foreman. For that, the two-fluoroscopic technique can provide an immediate AP view to prevent or detect earlier paravertebral cement leakages, if any.

Gangi et al. [2] reported that using combined CT and fluoroscopic guidance, 868 vertebroplasties were performed in which there were 15 (1.7%) cases with epidural cement leakage and 15 (1.7%) cases with significant leakage into the disc. Compared with Gangi's study, the incidence of cement leakage was higher in our study. However, most cases with cement leakage in the current study presented leakage into adjacent intervertebral discs except that one revealed epidural cement leakage (Case no. 14 in Group 2). There are other additional advantages of using the two-fluoroscope technique as followings. First, once the two fluoroscopes have been settled down, a well-trained staff is not needed to rotate the C-arm to obtain different radiographic views. The surgeon can simply take the radiographs by stepping on the switch. Second, the AP fluoroscope can precisely take a true AP view by tilting the C-arm with the radiation beam parallel to the coronal plane of the interesting vertebra, because the coronal plane of the low lumbar vertebrae are oblique and not vertical to the horizontal plane. Therefore, the AP fluoroscope with C-arm tilted adequately can take the true AP view in low lumbar vertebroplasty to show the full disc height and detect cement leakage into disc frankly.

In spite of the promising preliminary result, this study has some shortcomings need to be mentioned. First, we acknowledge that case numbers in our study was limited and was a retrospective design. A randomized clinical trial with more cases included is urged. Second, the radiation dose from the two-fluoroscopic technique causes considerable concern. It urges another study on the issue of radiation dose.

## Conclusion

This study demonstrates that the two-fluoroscopic technique provides concurrent, real-time AP and lateral radiographic views and can reduce the operation time. There is no symptomatic cement leakage or wound infection noted in this investigation. The two-fluoroscopic technique did not require a complex manpower organization and has been proved to be a safe and effective technique for PV.

## Competing interests

The authors declare that they have no competing interests.

## Authors' contributions

Y–YL conceived of the study, performed all operations and drafted the manuscript. T–JH participated in the design of the study and approved the version to be published. C–CC carried out the interviews for the patient satisfaction and analyzed radiographic measurements for cement leakage. RW–WH participated in the design of the study and coordination.

## Pre-publication history

The pre-publication history for this paper can be accessed here:


